# Overview of BioCreAtIvE: critical assessment of information extraction for biology

**DOI:** 10.1186/1471-2105-6-S1-S1

**Published:** 2005-05-24

**Authors:** Lynette Hirschman, Alexander Yeh, Christian Blaschke, Alfonso Valencia

**Affiliations:** 1 The MITRE Corporation, 202 Burlington Road, Bedford, MA 01730, USA; 2 Bioalma, Ronda de Poniente, 4 – 2nd floor, Unit C-D 28760 Tres Cantos, Madrid, Spain; 3 Protein Design Group, National Center of Biotechnology, CNB-CSIC, Cantoblanco, E-28049 Madrid, Spain

## Abstract

**Background:**

The goal of the first BioCreAtIvE challenge (Critical Assessment of Information Extraction in Biology) was to provide a set of common evaluation tasks to assess the state of the art for text mining applied to biological problems. The results were presented in a workshop held in Granada, Spain March 28–31, 2004. The articles collected in this *BMC Bioinformatics *supplement entitled "A critical assessment of text mining methods in molecular biology" describe the BioCreAtIvE tasks, systems, results and their independent evaluation.

**Results:**

BioCreAtIvE focused on two tasks. The first dealt with extraction of gene or protein names from text, and their mapping into standardized gene identifiers for three model organism databases (fly, mouse, yeast). The second task addressed issues of functional annotation, requiring systems to identify specific text passages that supported Gene Ontology annotations for specific proteins, given full text articles.

**Conclusion:**

The first BioCreAtIvE assessment achieved a high level of international participation (27 groups from 10 countries). The assessment provided state-of-the-art performance results for a basic task (gene name finding and normalization), where the best systems achieved a balanced 80% precision / recall or better, which potentially makes them suitable for real applications in biology. The results for the advanced task (functional annotation from free text) were significantly lower, demonstrating the current limitations of text-mining approaches where knowledge extrapolation and interpretation are required. In addition, an important contribution of BioCreAtIvE has been the creation and release of training and test data sets for both tasks. There are 22 articles in this special issue, including six that provide analyses of results or data quality for the data sets, including a novel inter-annotator consistency assessment for the test set used in task 2.

## Introduction

We provide here an overview of BioCreAtIvE, as an introduction to the papers included in this special issue. The article describes our motivation for creating BioCreAtIvE, our emphasis on applications of biological importance, and our commitment to building an infrastructure for critical assessment of text mining, including assessment of the quality of the training and test data.

BioCreAtIvE focused on two tasks. The first dealt with extraction of gene or protein names from text, and their mapping into standardized gene identifiers for three model organism databases (fly, mouse, yeast). The second task addressed issues of functional annotation, requiring systems to identify short text passages that supported Gene Ontology annotations for specific proteins, given full text articles.

There are a total of 22 articles in the special issue: 14 articles related to the first task in BioCreAtIvE (divided into two subtasks) and 8 articles associated with the second task. The articles are listed in Table 1, along with authors, affiliations and task.

Task 1a focuses on extraction of gene mentions from single sentences in MEDLINE abstracts; there are 8 papers on task 1a, including an overview article [1], an article on the construction of the training and test data [2], and 6 articles describing specific system approaches [3-9].

Task 1b requires the generation of lists of unique gene identifiers for genes mentioned in abstracts of articles curated in one of three model organism databases. There are 6 papers for task 1b, including an overview [10], an article describing preparation of the test sets and inter-annotator agreement experiments [11], and four articles describing systems and results for task 1b [9,12-14].

Task 2 focused on identifying text passages in full text articles that provide evidence for GO annotations about a particular protein. There are a total of eight articles for task 2: an overview article [15], an analysis of interannotator agreement by the EBI GOA annotation team [16], and six articles on the system approaches to task 2 [17-22]. In addition, the complete proceedings of the BioCreAtIvE workshop are available on line at  these include system descriptions for all of the 27 participating groups.

## Background

### Why evaluate?

Our goal in organizing BioCreAtIvE was to provide a systematic assessment of the state of the art for a set of "building block" biological tasks. There has been increased activity in the field of text mining and information extraction applied to the biological literature. However, each group has tackled a different problem and reported on a different data set [23]. With BioCreAtIvE, our goal was to assemble a suite of tasks that would:

• Attract researchers from both natural language processing and bioinformatics;

• Address problems of importance to the biology and bioinformatics community;

• Create legacy training and test data suites that could be used for development and benchmarking of future applications.

• Permit the assessment of the state of the art on real biological tasks.

We chose to frame these tasks in terms of aids for the curation of biological databases

This built on earlier work for the KDD Challenge Cup, one of the first challenge evaluations in text mining for biology [24]. The KDD Cup also focused on a task related to the curation of biological literature, namely the identification of articles containing experimental evidence for gene products for Flybase [25].

In creating this framework for assessment of text mining in biology, we were able to build on related research from both the biology and the natural language communities. The biology/bioinformatics community has created a number of successful evaluations, including CASP (Critical Assessment of Techniques for Protein Structure Prediction) [26]. In the computer science/text processing community, there have been two major models for evaluation. The first was the series of seven Message Understanding Conferences or MUCs held in the 1990's [27]; these focused on extraction of "named entities" (person, organization, location) and more complex relations and events from news articles. The second related evaluation is the recently introduced Genomics track [28] of annual Text Retrieval Conferences (TREC) [29].

### Choice of evaluation task

In designing the tasks for BioCreAtIvE, we were motivated by several factors: first, the need to define meaningful biological applications; second, the availability of training and "gold standard" test data; third, the need for a simple, realistic evaluation procedure; and fourth, the desire to bring together participants from fields such as natural language processing and text mining, as well as from bioinformatics.

By choosing tasks related to the curation process of some of the major biological databases, we guaranteed that the tasks would have biological relevance, since these are tasks that are presently performed by expert human curators. A focus on curation also made it possible to involve human experts, who have in-depth knowledge about the problems of annotation and the handling of biological information. This also meant that there would be "gold standard" annotated data available: annotations produced by expert curators that could be used to as training data for system development and as an evaluation standard for the blind test data.

**Figure 1:**
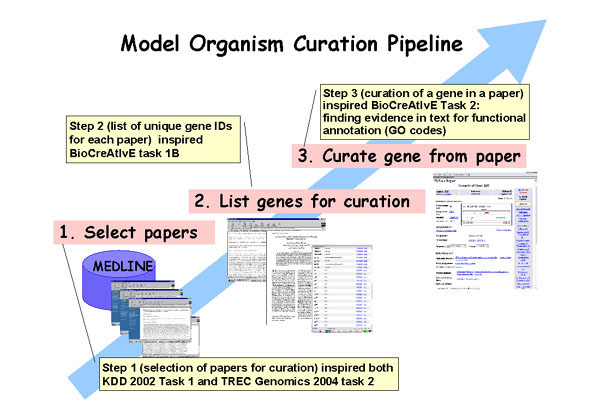
Curation pipeline for a model organism database.

Figure 1 shows a typical pipeline for biological curation, in this case represented by the curation of GO annotations. It begins with the selection of relevant articles from the literature, e.g., identifying all papers that discuss a certain set of genes for a given organism. In general, there is an additional requirement that the papers contain "curatable" information – experimental findings on a particular gene or gene product. This document retrieval task was the inspiration for the KDD 2002 Challenge Cup Task 1 [24]; it also inspired the categorization task in TREC Genomics 2004 [28]. This initial step was not represented in the BioCreAtIvE tasks.

The second step in the curation pipeline involves listing the genes or gene products that have sufficient information in the article to warrant curation. The list is given as unique gene identifiers for the genes of the particular model organism. This task formed the basis for task 1, gene name extraction and normalization (specifically, task 1b, listing the unique gene identifiers in a paper).

The third step involves the actual curation of a gene or gene product: namely, the assignment of properties to the genes and gene products identified in the previous step, based on experimental findings reported in the literature. A major advance in recent years has been the adoption of a shared ontology across organisms, namely the Gene Ontology or GO [30]. GO provides ontologies that allow annotators to describe molecular function, biological process and cellular localization of genes and gene products; there are now some 30 participating databases and/or organisms using GO. This curation step forms the basis for BioCreAtIvE task 2, assigning functional annotation for specific genes based on evidence provided in the literature.

In the context of BioCreAtIvE, we chose these tasks to cover a wide range of complexity, from the relatively easy task of listing the genes or gene products, representing a direct information extraction problem, to the very demanding GO annotation task, which requires additional interpretation of the meaning of the annotations in their ontological and biological contexts.

## Results

### Task 1

Task 1 was divided into two sub-tasks, reflecting different sources of data. Task 1a focused on the identification of gene or protein names in running text. The data for this task was provided by Lorrie Tanabe and John Wilbur (NCBI) [2] and was derived from annotation of single sentences taken from MEDLINE abstracts. This task was very close to the "named entity tagging" task that has been used extensively in the natural language processing community. This made it easy for many groups to participate whose main expertise was in natural language processing – this was the most heavily subscribed BioCreAtIvE task, with 15 teams participating.

An example sentence is shown below:

Furthermore, as in the human gene, the 3' end of the Cacna1f gene maps within 5 kb of the 5' end of the mouse synaptophysin gene in a region orthologous to Xp11/23.

In this example, the system must identify the gene/protein names *Cacna1f gene *(or *Cacna1f*) and *mouse synaptophysin gene *(or minimally, *synaptophysin*). However, a phrase like "the human gene" is not marked because it is not the name of a particular gene. The answer key provides for alternative forms, e.g., *Cacna1f gene *or *Cacna1f*.

Participants were given 10,000 annotated training sentences and were tested on an additional 5000 blind test sentences. The main findings from task 1a were that four different teams, using techniques such as Hidden Markov Models and Support Vector Machines, were able to achieve F-measures over 0.80 (F-measure is the harmonic mean of precision and recall). This is somewhat lower than figures for similar tasks from the news wire domain. For example, extraction of organization names has been done at over 0.90 F-measure. The article by Yeh et al [1] provides an analysis of these differences, attributing about half the difference in F-measure to the fact that systems show lower performance for longer names (also noted in [3]), and the distribution of gene and protein names is skewed towards longer names than seen for organization names.

Data preparation for task 1a [1,2] had several interesting features. In particular, the data were annotated by biologists, without explicit annotation guidelines. This is a novel approach to annotation: annotation of named entities for news wire (e.g., person, organization, location, etc) for the Message Understanding Conference tasks required extensive multi-page annotation guidelines [27]. For task 1a, there were no systematic inter-annotator agreement studies carried out to assess the quality of the test data. However, some post-evaluation analysis indicated that there may have been inconsistencies in how compound terms were annotated, such as "Mek-Erk1/2 pathway".

These inconsistencies made it difficult to learn generalizations from the training data, thus reducing scores; this may also account for some of the discrepancy between performance on the gene/protein name extraction task, compared to the news wire tasks.

Task 1a was viewed as a "building block" task – a task that could be treated as a natural language processing task that required no significant biological expertise. It also constitutes a first step for more complex tasks, such as gene name normalization (task 1b) or functional annotation of genes (task 2).

Task 1b focused on creating normalized gene lists; this is a task that is currently performed (manually) by curators for various model organism databases. This meant that there was a readily available data set for both training and testing. We chose three model organism databases (fly [25], mouse [31], yeast [32]) as sources of gene lists associated with papers. Our goal in choosing several model organisms was to encourage approaches that could be readily applied to different vocabularies.

We were committed to providing large training and test sets for this task. Due to the difficulties of obtaining large quantities of full text articles, we chose to provide only the abstracts of articles from MEDLINE for the evaluation. This meant that we had to edit the gene lists to make them correspond to genes mentioned in the abstract, rather than all the genes curated in the full text article. We developed a procedure to automatically remove genes not found in the abstract and were able to provide a large quantity of "noisy" training data for the three organisms, together with small collections of carefully corrected development and test data [11]. We estimated the quality of the noisy training data for the three organisms. Yeast training data quality appeared to be quite good (precision 0.99, recall 0. 86); fly training data was a little noisier (precision 0.92, recall 0.86); and mouse training data had poor recall (precision 0.99, recall 0.55). We also provided synonym lists for each organism, consisting of the unique gene identifier and its alternate names, as listed in the resources provided by each model organism database.

**Figure 2:**
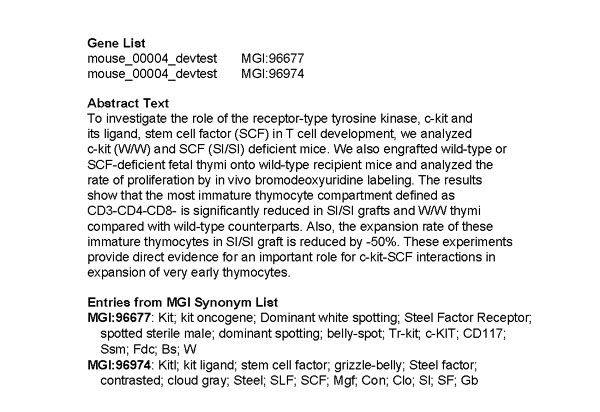
Sample abstract with unique gene identifiers, plus excerpt from lexicon.

Figure 2 shows a sample abstract with the associated unique gene identifiers, plus an excerpt from the lexicon, showing the many alternate names associated with genes. Although genes may be mentioned more than once in an abstract, the gene list consists of the set of *unique *mouse genes mentioned in the abstract.

There were eight groups participating in task 1b. The results [10] varied considerably, from a high for yeast of 0.92 F-measure, to somewhat lower scores for fly (high F-measure of 0.82) and mouse (high F-measure of 0.79). Our analysis [10] showed that the differences among organisms could be attributed to a variety of factors, including extensive ambiguity in names and overlap of gene names with English terms (fly); complex multi-word gene names (mouse); and quality of the training data, especially for mouse, where recall on the training data was estimated at 55%.

These results lead us to believe that tools for automated gene name identification and normalization may be ready to be incorporated into the curation process, at least where organism nomenclature is highly regular, such as yeast, and authors adhere to the model organism database conventions in the literature. However in many cases, the real task is even more complicated, for example, when papers for several organisms are simultaneously analyzed, since the same names are used for different genes in different species.

### Task 2

Task 2 focused on the automatic assignment of GO annotations to human proteins, based on full text articles. There were several parts to task 2, corresponding to ascending degrees of difficulty [15]. For task 2, the organizers made a conscious decision to provide data "as is" to reflect the realities of a biological application. The training set consisted of around 800 full text journal articles and their associated annotations (protein and GO code) taken from GOA . These were released to participants with no further annotations – that is, it was left to participants to determine the evidence passages that supported the GO annotations. The test set consisted of approximately 200 articles that were curated by the GOA team specifically for the assessment; these were not released until after the assessment was complete, to keep the data blind. In contrast to task 1, the participants also had to find their own lexical resources, such as synonyms for GO terms as well as protein name synonyms.

The input for task 2.1 consisted of triples made up of a pointer to a full text article, a protein (SWISS-PROT ID) and a GO code. The task was to return a short text passage providing evidence for the GO code assigned to that protein. Ideally, the text passage was to contain a mention of the protein and the evidence for the GO code assignment. These passages were judged for correctness by expert curators from the EBI GOA team [16]. There were approximately 1000 triples presented to the systems for task 2.1.

**Figure 3:**
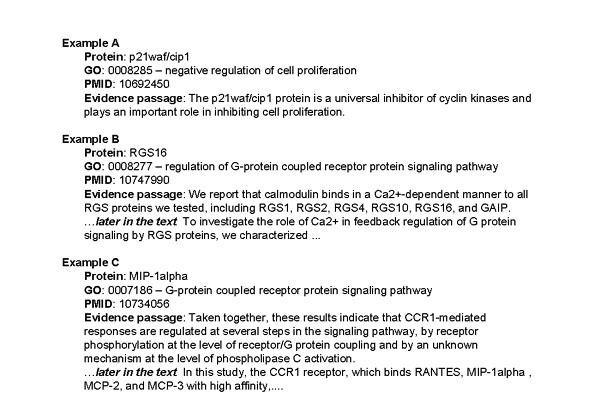
SWISS-PROT ID, GO code, PubMed ID triple and the corresponding evidence passage from the article (3 examples).

Figure 3 shows three examples of triples and the corresponding text passages. Example 3a is relatively easy, because both the protein and a description of the function or process appear in a single sentence. Figure 3b illustrates why this task is hard. The first sentence provides the information that the protein of interest (RGS16) is an RGS protein: "We report that calmodulin binds in a Ca2+-dependent manner to all RGS proteins we tested, including RGS1, RGS2, RGS4, RGS10, RGS16, and GAIP...". This knowledge then makes it possible to identify evidence in a later sentence that supports the GO annotation (regulation of G-protein coupled receptor protein signaling pathway): "To investigate the role of Ca2+ in feedback regulation of G protein signaling by RGS proteins, we characterized..." Finally, Figure 3c is harder still, requiring some reasoning to determine evidence for the annotation of MIP-1alpha. The first sentence establishes that CCR1 is related to a G-protein coupled receptor pathway and the second sentence states that MIP-1alpha binds to this receptor, which supports the deduction that it is also related to this process.

As these examples show, task 2.1 was a very difficult task. It required not only name extraction and normalization for proteins (as in task 1), but also the ability to recognize different ways of phrasing GO terms – without any training data. In addition, it also required an understanding of the connections among multiple sentences in an article, including the handling of co-reference and reasoning about connections among entities mentioned in those sentences.

We found it encouraging that several systems were able to return over 300 answers (out of approximately 1000 possible) that were judged correct by the assessors. The different systems used a wide range of strategies. For example, several systems returned answers only where the evidence was very strong; these systems returned very few answers but with a higher proportion correct.

Task 2.2 was more difficult still: for this task, the test data consisted of triples of text, protein, and number of GO codes (but not the actual GO codes). The systems were required to return not only evidence passages as before, but also GO code assignments for the protein. The performance dropped by roughly a factor of two from performance on task 2.1.

Overall, the performance on task 2 is not surprising. Task 2.1 involves three subtasks: identification of protein, identification of GO term, and correct association of these two. Identification of the protein mention in text would be roughly comparable to task 1b, and we would expect the best systems to achieve between 70–80% accuracy. Identification of mentions of GO terms would be substantially harder. Analysis of the results [15] revealed that GO terms for cellular location turned out to be easier than terms for biological process. This may be related to the fact that terms for cellular location were shorter and more "concrete" than terms describing abstract complex relations such as biological process. By contrast, terms for biological process are abstract and complex, e.g., "cytokine and chemokine mediated signaling pathway". We would expect performance on GO term identification to be significantly lower than performance on protein name identification. Furthermore, finding the correct association between protein and GO annotation, especially where the association requires integration of information across multiple sentences, constitutes an additional difficulty. If each of these three steps were accomplished at around 70% accuracy, the final outcome would be close to the observed overall accuracy for task 2.1 of about 30%.

The results for task 2 demonstrate that current systems are not yet able to produce satisfactory results for the extraction of biological information, especially where it requires complex extrapolation and integration. However, this assessment represents an important baseline. We expect that these results will improve with the availability of training data generated from the task 2.1 and 2.2 submissions. Also, creation of lexical resources for GO terms and paraphrases should make it easier to recognize GO terms in text.

## Methods

It is critical to evaluate an evaluation. The success of an evaluation can be gauged by several criteria:

• The level of participation: did the evaluation attract good researchers from diverse groups and backgrounds?

• The results: was the task sufficiently challenging, but not too easy, or too hard?

• The research: does the task raise important and interesting research questions?

• The relevance of the application: does the evaluation task have applicability to some application that users care about?

• The data: were there sufficient amounts of training and test data? Was the quality of the data good enough? Will these resources be available to the larger research community after the evaluation, for further benchmarking?

• Repeatability: Would people want to do this again? Is it easy to do again?

Overall, we believe that the BioCreAtIvE evaluation was a major success along all of these dimensions.

### Participation

We attracted 27 groups from ten countries, including participants from some of the major groups involved in information extraction in biology. These included bioinformatics groups as well as computational linguistics groups, as well as participation at the BioCreAtIvE workshop from two major biological database groups: EBI SWISS-PROT GOA team (Apweiler, Camon and Lee) and MGI (Blake).

### Results

The results were encouraging for task 1, with high scores in the range of 0.8 to 0.9 F-measure, depending on the sub-task. For task 2, the results created a good baseline for future experimentation. Functional annotation is a difficult task for humans, as the inter-annotator agreement experiments in [16] showed. The results appearing in this special issue will set the bar for future experimentation and progress in this field. As discussed in [15], it is likely that future systems can improve their results substantially by training with the annotated results provided in this first BioCreAtIvE challenge.

### Research

The tasks raised interesting questions of biological relevance. Task 1a (gene name tagging) provided a good comparison point to comparable tasks in other domains. Task 1b (normalized gene lists for abstracts from three model organism databases) raised the question of how to build a system that could be quickly adapted to new vocabularies and different lexical resources. Task 2 represents an ambitious "end goal" for text mining, requiring the ability to map complex concepts expressed in free text to ontological concepts from GO. The semantic distance between a simple concept name in GO and its expression in text made this particularly challenging.

### Relevance

Tasks 1b and 2 were of clear biological relevance. Task 1b was designed to emulate a curation task that is now done manually and the data were derived from biological databases. For task 2, this task was of such real relevance for curation that we were able to recruit expert curators to spend several person-months of labor assessing results for task 2.

### Data

We used three different data sets. For task 1a, the data were prepared in a novel way (with no explicit guidelines). There were sufficient training and development test sets, and there is sufficient test data set aside for another round of evaluation. For task 1b, we were able to use "noisy" training data, though the noisy data may have imposed limitations on system performance [10]. And the difficulties of achieving reliable interannotator agreement were greater than we expected [11]. Finally, for task 2, we were fortunate to receive high quality expert judgements for the vast majority of submissions. These data judgements will now form a valuable annotated training set for future evaluations. For both tasks, the training and test data are now available for other groups to use in further experiments (see  and also ).

In our search for appropriate data sets, we have found that there are difficult trade-offs between providing good datasets for research and providing challenge problems that reflect the reality (and messiness) of real biological applications. The ideal data set for research would consist of a large quantity of expert-annotated data, done on a real biological task, making use of available data sets.

In reality, there are a number of difficulties. First, although many tasks require full text articles, it is difficult to find large collections of full text articles that are freely available; the choices are often between large quantities of abstracts or much smaller quantities of full text. This was the trade-off made for BioCreAtIvE task 1. Second, there is the issue of realism. To make a good assessment, the task must be well defined, but this may make the assessment task less realistic. BioCreAtIvE task 1b attempted to provide a degree of realism by using three data sets from three different model organisms. However, a more realistic task would have been to associate gene names with organisms with no prior separation by organism. We also hoped to raise the issue of system adaptation: how to tailor a system to slightly different applications, given different sets of lexical resources. However, we observed that most groups built different systems for each model organism, in an attempt to achieve the best performance for each specific data set. Finally, there is the issue of training data. For research, the more carefully curated training data, the better. But it can be difficult to provide training data for a real task that hasn't been automated. Currently curators do not capture the evidence passages in a paper as they curate, partly because there are no easy-to-use tools to support this. As a result, all that is visible at the end of the curation process are the annotations at the level of the entire paper, and there are no sets of fine-grained curator annotations to use as training data for task 2. This meant that task 2 was realistic – but more difficult because of lack of training data; for a follow-on evaluation, however, the assessed submissions by the participants will be available and can be used to derive fine-grained judgements about evidence passages.

### Repeatability

The participants at the workshop seemed eager to repeat the evaluation; in addition, we have received a constant stream of requests for the various data sets. We are now searching for the financial support necessary for a second round of BioCreAtIvE. The cost of repeating the different parts of the evaluation will vary. For example, there is an additional blind test set for task 1a available with no further work. For task 2, we will need to find a way to reduce the time that curators spent assessing the correctness of the submissions. There are a number of suggestions for this in [16].

## Discussion

This special issue represents a major step forward in tracking the progress of text mining applied to pressing biological information needs. The rate of accumulation of "raw data" (genome sequences, results of high-throughput experiments) is growing rapidly. Biological databases are also proliferating to organize these datasets into structures amenable to further computation. A major function of these databases is to associate the biological building blocks (sequence data, genes, proteins) with results from the published literature via ontologies or controlled vocabularies. This is currently an expensive and slow manual operation. To keep up with this deluge of data, it will be necessary to rely increasingly on automated aids to speed up this process. The BioCreAtIvE assessment and workshop activities constitute an important first step in creating an infrastructure and building together a multi-disciplinary community to tackle these urgent problems.

## Authors' contributions

The idea for BioCreAtIvE came out of discussions between AV and LH over a period of several years. The execution of BioCreAtIvE was a joint activity of the MITRE team (LH, ASY for task 1), and the CNB/CSIC team (CB, AV for task 2). CB was responsible for organizing the BioCreAtIvE workshop under sponsorship of EMBO, and for the execution of task 2. AV is leader of the Protein Design Group at CNB/CSIC and was responsible for the creation of task 2 and the involvement of the EBI SWISS-PROT team in the assessment of that task. LH heads the MITRE Biotechnology effort and was responsible for organizing BioCreAtIvE task 1, and specifically responsible for the oversight and analysis of task 1b. ASY was responsible for running task 1a of BioCreAtIvE and for the analysis of task 1a results.

## Figures and Tables

**Table 1:** List of papers in the special issue on BioCreAtIvE

**Ref**	**Task**	**Authors**	**Institution**	**Country**	**Title**
	all	Hirschman, L., Yeh, A., Blaschke, C., Valencia, A.	MITRE BioAlma National Center of Biotechnology, CNB-CSIC	US Spain	Overview of BioCreAtIvE: Critical Assessment of Information Extraction in Biology

1	1A	Yeh, A., Morgan, A., Colosimo, M. Hirschman, L.	MITRE	US	BioCreAtIvE task 1A: gene mention finding evaluation

2	1A	Tanabe, L., Xie, T.N. Thom, L.H., Matten, W. Wilbur, W.J.	NIH Consolidated Safety NIH	US	Genetag: A Tagged Corpus for Gene/Protein Named Entity Recognition

3	1A	Kinoshita, S.	University of Colorado School of Medicine and Fujitsu	US/Japan	BioCreAtIvE Task 1A: Entity Identification with a Stochastic Tagger
		Cohen, K.B., Ogren, P.V.	University of Colorado School of Medicine	US	
		Hunter, L.	Center for Computational Pharmacology, University of Colorado School of Medicine	US	

4	1A	Finkel, J. Dingare, S. Manning, C.D. Nissim, M., Alex, B., Grover, C.	Stanford University University of Edinburgh Stanford University University of Edinburgh	US UK US UK UK	Exploring the Boundaries: Gene and Protein Identification in Biomedical Text

5	1A	McDonald, R., Pereira, F.	University of Pennsylvania	US	Identifying Gene and Protein Mentions in Text using Conditional Random Fields

6	1A	Zhou, G.D.	Institute for Infocomm Research and the University of Singapore	Singapore	Recognition of Protein/Gene Names from Text using an Ensemble of Classifiers
		Shen, D., Zhang, J., Su, J., Tan, S.H.	Institute for Infocomm Research		

7	1A	Mitsumori, T., Fation, S.	Nara Institute of Science and Technology	Japan	Gene/Protein Name Recognition based on Support Vector Machine using Dictionary as Features
		Murata, M.	National Institute of Information and Communications Technology		
		Doi, K., Doi, H.	Nara Institute of Science and Technology		

8	1A	Hakenberg, J., Bickel, S., Plake, C., Brefeld, U., Zahn, H., Faulstich, L., Leser, U., Scheffer, T.	Humboldt-Universität zu Berlin, Computer Science Department	Germany	Systematic Feature Evaluation for Gene Name Recognition

9	1A, 1B	Tamames, J.	BioAlma	Spain	Text Detective: A Rule-based System for Gene Annotation in Biomedical Texts

10	1B	Hirschman, L., Colosimo, M., Morgan, A., Yeh, A.	MITRE	US	Overview of BioCreAtIvE task 1B: Normalized Gene Lists

11	1B	Colosimo, M., Morgan, A., Yeh, A., Colombe, J., Hirschman, L.	MITRE	US	Data Preparation and Interannotator Agreement: BioCreAtIvE Task 1B

12	1B	Crim, J., McDonald, R., Pereira, F.	University of Pennsylvania	US	Automatically Annotating Documents with Normalized Gene Lists

13	1B	Hanisch, D. Fundel, K. Mivissen, H.T. Zimmer, R. Fluck, J.	Fraunhofer Institute SCAI Institute for Informatics Fraunhofer Institute SCAI Institute for Informatics Fraunhofer Institute SCAI	Germany	ProMiner: Rule-based Protein and Gene Entity Recognition

14	1B	Fundel, K., Guttler, D., Zimmer, R., Apostolakis, J	Institute für Informatik	Germany	A Simple Approach for Protein Name Identification: Prospects and Limits

15	2	Blaschke, C. Krallinger, M., Leon, E.A., Valencia, A.	BioAlma National Center of Biotechnology, CNB-CSIC	Spain	Evaluation of BioCreAtIvE assessment of task 2

16	2	Camon, E.B., Barrell, D.G., Dimmer, E.C., Lee, V., Magrane, M., Maslen, J., Binns, D., Apweiler, R.	European Molecular Biology Laboratory-European Bioinformatics Institute	UK	An evaluation of GO annotation retrieval for BioCreAtIvE and GOA

17	2	Ray, S., Craven, M.	University of Wisconsin	US	Learning Statistical Models for Annotating Proteins with Function Information using Biomedical Text

18	2	Krallinger, M., Padron, M., Valencia, A.	National Center of Biotechnology, CNB-CSIC	Spain	A Sentence Sliding Window Approach to Extract Protein Annotations from Biomedical Articles

19	2	Verspoor, K., Cohn, J., Joslyn, C., Mniszewski, S., Rechsteiner, A.	Los Alamos National Lab	US	Protein Annotation as Term Categorization in the Gene Ontology using Word Proximity Networks
		Rocha, L., Simas, T.	Indiana University	US	

20	2	Couto, F., Silva, M.	Faculdade de Ci¸ncias da Universidade de Lisboa, Lisbon, Portugal	Portugal	Finding Genomic Ontology Terms in Unstructured Text
		Coutinho, P.	Architecture et Fonction des Macromolécules Biologiques, CNRS	France	

21	2	Rice, S., Nenadic, G., Stapley, G.	University of Manchester	UK	Mining Protein Function from Text using Term-based Support Vector Machines

22	2	Ehrler, F.	University of Geneva and University Hospital of Geneva	Switzerland	Data-poor Categorization and Passage Retrieval for Gene Ontology Annotation in Swiss-Prot
		Jimeno, A.	University Hospital of Geneva and CERN, Geneva		
		Ruch, P	University Hospital of Geneva		

## References

[B1] Yeh AS, Morgan A, Colosimo M, Hirschman L (2005). BioCreAtIvE task 1A: gene mention finding evaluation. BMC Bioinformatics.

[B2] Tanabe L, Xie N, Thom LH, Matten W, Wilbur WJ (2005). GENETAG: A Tagged Corpus for Gene/Protein Named Entity Recognition. BMC Bioinformatics.

[B3] Kinoshita S, Cohen KB, Ogren PV, Hunter L (2005). BioCreAtIvE Task 1A: Entity Identification with a Stochastic Tagger. BMC Bioinformatics.

[B4] Finkel J, Dingare S, Manning CD, Nissim M, Alex B, Grover C (2005). Exploring the Boundaries: Gene and Protein Identification in Biomedical Text. BMC Bioinformatics.

[B5] McDonald R, Pereira F (2005). Identifying Gene and Protein Mentions in Text using Conditional Random Fields. BMC Bioinformatics.

[B6] Zhou GD, Shen D, Zhang J, Su J, Tan SH (2005). Recognition of Protein/Gene Names from Text using an Ensemble of Classifiers. BMC Bioinformatics.

[B7] Mitsumori T, Fation S, Murata M, Doi K, Doi H (2005). Gene/Protein Name Recognition based on Support Vector Machine using Dictionary as Features. BMC Bioinformatics.

[B8] Hakenberg J, Bickel S, Plake C, Brefeld U, Zahn H, Faulstich L, Leser U, Scheffer T (2005). Systematic Feature Evaluation for Gene Name Recognition. BMC Bioinformatics.

[B9] Tamames J Text Detective: A Rule-based System for Gene Annotation in Biomedical Texts. BMC Bioinformatics.

[B10] Hirschman L, Colosimo M, Morgan A, Yeh A (2005). Overview of BioCreAtIvE task 1B: Normalized Gene Lists. BMC Bioinformatics.

[B11] Colosimo M, Morgan A, Yeh A, Colombe J, Hirschman L (2005). Data Preparation and Interannotator Agreement: BioCreAtIvE Task 1B. BMC Bioinformatics.

[B12] Crim J, McDonald R, Pereira F (2005). Automatically Annotating Documents with Normalized Gene Lists. BMC Bioinformatics.

[B13] Hanisch D, Fundel K, Mevissen HT, Zimmer R, Fluck J (2005). ProMiner: Rule-based Protein and Gene Entity Recognition. BMC Bioinformatics.

[B14] Fundel K, Guttler D, Zimmer R, Apostolakis J (2005). A Simple Approach for Protein Name Identification: Prospects and Limits. BMC Bioinformatics.

[B15] Blaschke C, Krallinger M, Leon EA, Valencia A (2005). Evaluation of BioCreAtIvE assessment of task 2. BMC Bioinformatics.

[B16] Camon EB, Barrell DG, Dimmer EC, Lee V, Magrane M, Maslen J, Binns D, Apweiler R (2005). An evaluation of GO annotation retrieval for BioCreAtIvE and GOA. BMC Bioinformatics.

[B17] Ray S, Craven M (2005). Learning Statistical Models for Annotating Proteins with Function Information using Biomedical Text. BMC Bioinformatics.

[B18] Krallinger M, Padron M, Valencia A (2005). A Sentence Sliding Window Approach to Extract Protein Annotations from Biomedical Articles. BMC Bioinformatics.

[B19] Verspoor K, Cohn J, Joslyn C, Mniszewski S, Rechsteiner A, Rocha L, Simas T (2005). Protein Annotation as Term Categorization in the Gene Ontology using Word Proximity Networks. BMC Bioinformatics.

[B20] Couto F, Silva M, Coutinho P (2005). Finding Genomic Ontology Terms in Unstructured Text. BMC Bioinformatics.

[B21] Rice S, Nenadic G, Stapley G (2005). Mining Protein Functions from Text using Term-based Support Vector Machines. BMC Bioinformatics.

[B22] Ehrler F, Jimeno A, Ruch P (2005). Data-poor Categorization and Passage Retrieval for Gene Ontology Annotation in Swiss-Prot. BMC Bioinformatics.

[B23] Hirschman L, Park JC, Tsujii J, Wong L, Wu CH (2002). Accomplishments and challenges in literature data mining for biology. Bioinformatics.

[B24] Yeh AS, Hirschman L, Morgan AA (2003). The evaluation of text data mining for database curation: lessons learned from the KDD challenge cup. Bioinformatics.

[B25] The FlyBase Database:. http://flybase.org/.

[B26] CASP: Critical Assessment of Techniques for Protein Structure Predication:. http://predictioncenter.llnl.gov/casp6/Casp6.html.

[B27] MUC-7: Seventh Message Understanding Conference. http://www.itl.nist.gov/iaui/894.02/related_projects/muc/proceedings/muc_7_toc.html.

[B28] Hersh WR, Bhuptiraju RT, Johnson P, Cohen AM, Kraemer DF (2005). TREC 2004 Genomics Track Overview. Proc of TREC 2004, to appear as NIST Special Publication.

[B29] Text REtrieval Conference. http://trec.nist.gov/.

[B30] The Gene Ontology Consortium (2000). Gene Ontology: tool for the unification of biology. Nature Genet.

[B31] The Mouse Genome Database. http://www.informatics.jax.org.

[B32] Saccharomyces Genome Database. http://www.yeastgenome.org.

